# Supplementary material for: Knowledge, and attitude of service user of intermittent preventive treatment of malaria in pregnancy using sulfadoxine pyrimethamine in the Volta Region of Ghana
Source: PLoS One. 2024 Sep 6;19(9):e0309340. doi: 10.1371/journal.pone.0309340 (PMC11379158; doi:10.1371/journal.pone.0309340)
Supplement: S1 Checklist — (DOCX) [file pone.0309340.s001.docx]

## **Appendix 3: Questionnaire for Service Users (Survey)**

Good morning/Afternoon. My name is ***Livingstone Asem*** a PhD. Candidate working on the ***Assessment of implementation effectiveness of intermittent preventive treatment of malaria in pregnancy using sulfadoxine-pyrimethamine***. Your genuine response is very much needed for better improvement of the implementation of intermittent preventive treatment of malaria in pregnancy in the Volta Region. Your participation is completely voluntary and anonymous. You have the liberty to decide to contribute or withdraw at any time of the study. You are also assured of confidentiality. No information on you will be disclosed to anybody and your name will not in any way be linked to any information in this study. This is purely an academic exercise and as such all responses will be treated with the strictest confidence.

| QUESTIONAIRE NO._______________ | DATE OF INTERVIEW:  DD/MM/YYYY |
| --- | --- |
| DISTRICT:________________ | ENUMERATION AREA______________ |
| LOCATION:__________________ |  |

***Service Users***

| 1 | Age of Client | / |  |
| --- | --- | --- | --- |
| 2 | Marital Status | Married  Single  Divorced  Widowed  Cohabitation |  |
| 3 | Maternal Educational level | None  Primary  Middle/JSS/JHS  SSS/SHS  Tertiary/Higher |  |
| 4 | Maternal Religion | Catholic  Anglican/Methodist/Presby/EP  Pentecostal/Charismatic  Other Christian  Muslim  Traditionalist/Spiritualist  No religion  Others |  |
| 5 | Maternal Occupation | Farmer/Agriculture  Sales &Services/Trader  Skilled manual/Artisan  Unskilled manual  Professional/Technical/Managerial  Unemployed  Clarical  Others |  |
| 6 | Ethnic group | Akan  Ga/dangme  Ewe  Guan  Konkonba  Chorkorsi  Others |  |
| 7 | Partner Educational level (Husband) | None  Primary  Middle/JSS/JHS  SSS/SHS  Tertiary/Higher |  |
| 8 | Partner Occupation (Husband) | Farmer/Agriculture  Sales &Services/Trader  Skilled manual/Artisan  Unskilled manual  Professional/Technical/Managerial  Unemployed  Clerical  Others |  |
| 9 | Age of partner | …… |  |
| 10 | Duration of marriage ( years) | ……… |  |
| 11 | Monthly household Income | ……….. |  |
| 12 | Household Assets( chose as many as applied to client) | Radio  Color Television  Mobile phone  Non-mobile Telephone  Wall Clock  Generator  Computers/Tablet  Washing Machine  Video Deck/DVD/VCD/Decoder  Camera  Sewing Machine  Bed  Table  Internet Access  Cabinet/Cupboard  Refrigerator  Freezer  Means of transport(car, bicycle, motor, boat)  Ownership of Agric land  Ownership of farm animals |  |
| 13 | Means of transportation to nearest health facility( Clinic) | Walking  Vehicle  Motor Bike  Boat |  |
| 14 | Estimated time to health facility (Minutes) | …….. |  |
| 15 | Estimated distance to health facility(km) | ……... |  |
| 16 | Do you have Health Insurance | Yes  No |  |
| 17 | Are you covered/ active with NHIS | Yes  No |  |
| 18 | At what gestation age booking for ANC for last/current pregnancy ( in weeks) | …….. |  |
| 19 | At what gestational age did you first receive IPTp-SP for last pregnancy/Child ( in weeks) | ……. |  |
| 20 | Number of pregnancies (gravida) | …… |  |
| 21 | Parity (No. births) | …… |  |
| 22 | No. of live births | ……. |  |
| 23 | Number of ANC visits for current/last pregnancy ( Check from MCHRB if available) | …….. |  |
| 24 | How many doses of SP did your received for the last pregnancy ( Check from MCHRB if available) | 1  2  3  4  5 |  |
|  | **Knowledge of malaria and SP ( tick all correct answers that apply)**  ***In the past two years *** | |  |
| 25 | Cause of malaria | Eating Oily foods  Ant bite  mosquito bite  Dirty Environment  The Sun  Working around or with fire  Did not know |  |
| 26 | Signs and symptoms of malaria | Itching skin  High body temperature  Boils  Do not know |  |
| 27 | Effect of malaria on the pregnant woman | Can cause anaemia  Preterm labour/Miscarriage  Can cause deaths  Do not know |  |
| 28 | Effect of malaria on the unborn baby | Can cause spontaneous abortion  Can cause intra uterine death  Can cause low birth weight  Can cause prematurity  Do not know |  |
| 29 | What malaria prevention methods do you know | Sleeping under ITN  Use mosquito repellant  Wear protective clothing  Use SP  Do not know |  |
| 30 | Purpose of IPTp-SP is to prevent mother and baby from malaria. | Yes  No |  |
| 31 | Time to start IPTp-SP is on or before 16 weeks usually after quickening | Yes  No |  |
| 32 | How many tablets of SP per each dose should a woman received | One  Two  Three  Four |  |
| 33 | How many times should a pregnant woman receive this medicine (SP) during pregnancy | 3  4  5  Don’t know |  |
| 34 | What is the Interval between doses of SP | 1 month  2 months  1 week  2 weeks |  |
| 35 | How did you first get to know about IPTp-SP | Radio  TV  Health Worker  Other pregnant women  others |  |
|  | **Attitude on IPTp- SP uptake ( tick one correct answer only)**  ***In the past two years*** | |  |
| 36 | Is early ANC booking good for pregnant woman | Strongly Agree  Agree  Disagree  Strongly Disagree |  |
| 37 | Will you go for ANC booking before the 12^th^ weeks of pregnancy | Strongly Agree  Agree  Disagree  Strongly Disagree |  |
| 38 | I believe IPTp-SP is good for me and my foetus (baby) | Strongly Agree  Agree  Disagree  Strongly Disagree |  |
| 39 | ANC follow-up visit is good to monitor mothers and their foetus (baby) | Strongly Agree  Agree  Disagree  Strongly Disagree |  |
| 40 | The way health workers treat me will encourage me to attend next ANC (Attitude of health workers) | Strongly Agree  Agree  Neutral/Don’t Know  Disagree  Strongly Disagree |  |
| 41 | Was there delay in the waiting time/seen by health worker in the hospital/health facility | Strongly Agree  Agree  Neutral/Don’t Know  Disagree  Strongly Disagree |  |
| 42 | Was there privacy in the consulting room | Strongly Agree  Agree  Disagree  Strongly Disagree |  |
| 43 | Seek antenatal care regularly during pregnancy | Strongly Agree  Agree  Disagree  Strongly Disagree |  |
| 44 | Current pregnancy is planned? | Strongly Agree  Agree  Disagree  Strongly Disagree |  |
| 45 | Do you waiting for foetus to move before going for antenatal care | Strongly Agree  Agree  Disagree  Strongly Disagree |  |
| 46 | Are you able to meet the transportation cost to health facility | Strongly Agree  Agree  Disagree  Strongly Disagree |  |
| 47 | Feeling well and do not have any serious problem which need the attention of health staff | Strongly Agree  Agree  Disagree  Strongly Disagree |  |
| 48 | No power to make decision on your own. Husband/partner or family members have to make the decision for you to go for antenatal care | Strongly Agree  Agree  Disagree  Strongly Disagree |  |
| 49 | Husband/partner approval and support need before I attend antenatal care | Strongly Agree  Agree  Disagree  Strongly Disagree |  |
| 50 | Take five or more antenatal visits during pregnancy for IPTp-SP | Strongly Agree  Agree  Disagree  Strongly Disagree |  |
| 51 | The quality and content of service can be describe as good | Strongly Agree  Agree  Disagree  Strongly Disagree |  |
|  | **Personal experience of woman( service users)**  *In the past two years* | |  |
| 52 | Was SP given under Direct Observed Treatment (DOT) by provider | Given  Not given  Do not know/Can’t remember |  |
| 53 | Given prior information on drug by provider on purpose/importance | Given  Not given  Do not know/ Can’t remember |  |
| 54 | Given prior information on drug by provider on side-effects | Given  Not given  Do not know/ Can’t remember |  |
| 55 | Experience of drug side effects | Experience side effects  Side effects not experience  Do not know/ Can’t remember |  |
| 56 | Preference of DOT( will you prefer Direct Observed Therapy by provider) | Like taking SP under DOT  Does not like taking SP |  |
| 57 | Have ever taken SP drug home | Ever taken  Never taken |  |
| 58 | Have you ever had malaria Infection whiles on IPTp-SP | Had Malaria  Did not have malaria |  |
| 59 | Did the provider asked you to pay some amount of money for IPTp-SP services | Yes  No |  |
| 60 | Geo Points |  |  |
